# Relationship between thrombospondin-1, endostatin, angiopoietin-2, and coronary collateral development in patients with chronic total occlusion

**DOI:** 10.1097/MD.0000000000004524

**Published:** 2016-08-19

**Authors:** Qing Qin, Juying Qian, Jianying Ma, Lei Ge, Junbo Ge

**Affiliations:** Zhongshan Hospital, Fudan University, Shanghai Institute of Cardiovascular Disease, Shanghai, China..

**Keywords:** chronic total occlusion, coronary collateral, endostatin, thrombospondin-1

## Abstract

This study is aimed to investigate whether serum angiostatic factors (thrombospondin-1 [TSP-1] and endostatin) or angiogenic factors (angiopoietin-2 [Ang-2]) are related to coronary collateral vessel development in patients with chronic total occlusion (CTO).

A total of 149 patients were enrolled in the study, and 39 patients with coronary artery disease but without significant stenosis were included in control group. In 110 patients with CTO lesion, 79 with Rentrop grades 2 to 3 collaterals were grouped as good collateral, while 31 with Rentrop grades 0 to 1 collaterals were grouped as poor collateral. Serum TSP-1, endostatin, and Ang-2 levels were studied.

Serum endostatin level was significantly higher in poor collateral group compared with control group and good collateral group, respectively (96.2 ± 30.4 vs 77.8 ± 16.5 ng/mL, *P* = 0.007; 96.2 ± 30.4 vs 81.2 ± 30.4 ng/mL, *P* = 0.018). In multivariate analysis, decreased serum endostatin level was independently related to good coronary collateral development. Serum TSP-1 level was lower in patients with CTO compared with control group. However, no difference in TSP-1 level was detected between poor and good collateral group. The serum Ang-2 level did not show a significant difference among 3 groups.

Circulatory endostatin may be a useful biomarker for coronary collateral development and potential target for therapeutic angiogenesis in patients with CTO.

## 1 Introduction

Coronary collateral vessel development is a compensatory response to myocardial ischemia. In patients with severe coronary artery disease (CAD), such as chronic total occlusion (CTO), collateral vessels can salvage ischemic myocardium, help to preserve myocardial function, and exert a protective effect on prognosis.^[1,2]^ A variety of growth factors that act by stimulating endothelial and smooth muscle cell proliferation and migration, as well as substances that increase recruitment and activation of monocytes and stem cells have been demonstrated to stimulate angiogenesis and arteriogenesis.^[3]^ Induction of angiogenesis and arteriogenesis is a possible therapeutic strategy for promoting coronary collateral growth. Although animal studies showed promising results in delivering angiogenic growth factors (vascular endothelial growth factor [VEGF], fibroblast growth factor [FGF], hepatocyte growth factor [HGF], and angiopoietin-1 [Ang-1]) in ischemic myocardium,^[4–7]^ these preclinical studies did not translate into clinical success.^[8,9]^

Coronary collateral growth and development may be dependent on a balance between growth factors and growth inhibitors. Abundant evidence has been obtained in the role of angiogenic factors in ischemic heart disease. However, very few studies looked into the regulatory mechanism of antiangiogenic factors. In this study, we intend to explore the relationship between antiangiogenic proteins (thrombospondin-1 [TSP-1] and endostatin) and collateral formation in patients with CTO. Besides, angiogenic factor angiopoietin-2 (Ang-2) is also studied as its role in coronary collateral formation is not clear yet.

## 2 Method

### 2.1 Study population

**Figure 1:**
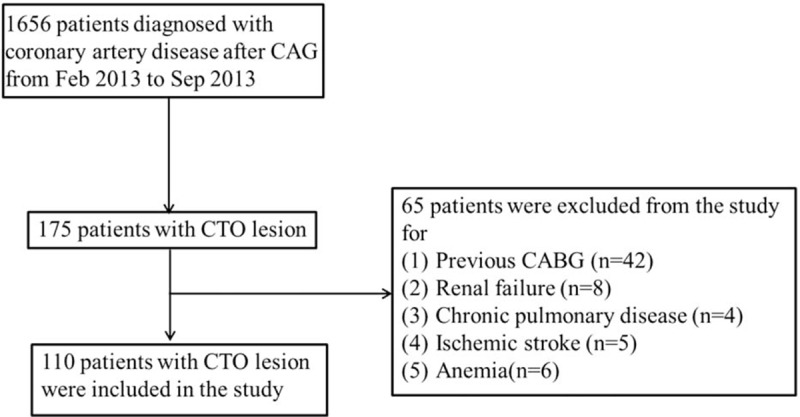
Flowchart of selecting CTO patients enrolled in the study. CABG = coronary artery bypass grafting, CAG = coronary angiography, CTO = chronic total occlusion.

Written informed consent was obtained from all participants or their legal representatives for use of their venous blood for measuring of serum TSP-1, endostatin, and Ang-2. Ethics approval was granted by the Human Research Ethics Committee of Zhongshan Hospital, Fudan University (Project no. B2013-014). Patients (n = 110) with CAD and at least 1 coronary artery occlusion of more than 3 months confirmed by coronary angiography (CAG) were prospectively enrolled in the study from February 2013 to September 2013. The control group was consisted of 39 patients who had atherosclerotic heart disease but no significant stenosis during the same period. The degree of the coronary artery stenosis was determined visually. Patients with chronic pulmonary disease, renal or hepatic failure, peripheral artery disease, ischemic stroke, tumor, anemia, and a previous coronary artery bypass graft operation were excluded from the study. Detailed data on selection of CTO patients are shown in Fig. 1.

### 2.2 Baseline characteristics

Information on traditional coronary risk factors was collected from medical records regarding age, gender, history of hypertension, diabetes mellitus, hyperlipidemia, smoking, drinking, and previous myocardial infarction (MI). Laboratory data including estimated glomerular filtration rate (eGFR), glucose, hemoglobin A1c (HbA1c), lipid profile, uric acid, N-terminal pro b-type natriuretic peptide (NT-proBNP), and left ventricular ejection fraction (LVEF) by ultrasonic cardiogram were also recorded.^[10]^

### 2.3 Coronary angiography and coronary collateral development grading

The standard selective coronary angiograms with at least 4 views of the left coronary system and 2 views of the right coronary artery (RCA) of the study patients were examined by 2 experienced interventional cardiologists who were blind to the study.^[10]^ CTO was defined as a complete interruption of coronary artery flow estimated to be at least 3 months duration by clinical, angiographic, or electrocardiographic criteria. Collateral filling of the recipient artery was assessed according to the Rentrop classification. Briefly, Rentrop grade is categorized as follows: 0—no filling of any collateral vessels, 1—filling of side branches of the epicardial segment, 2—partial filling of the epicardial artery by collateral vessels, and 3—complete filling of the epicardial artery by collateral vessels. If the patient had more than 1 CTO vessels, the highest Rentrop grade was recorded for analysis. Two independent observers, blinded to patient characteristics evaluated the collateral flow.^[11]^ Patients with grades 0 to 1 collateral development were regarded as poor collateral and patients with grades 2 to 3 collateral development were regarded as good collateral.

### 2.4 Blood samples

Venous blood samples for measurement of serum TSP-1, endostatin, and Ang-2 were collected just before the CAG in BD Vacutainer plastic K2EDTA tubes (BD Bioscience, Franklin Lakes, NJ, USA). Plasma was obtained by centrifugation at 1000 × *g* for 15 minutes within 30 minutes of collection. After an additional centrifugation step of the plasma at 10,000 × g for 10 minutes at 2°C to 8°C, samples were immediately stored at −80°C until analysis.^[11]^

### 2.5 Enzyme-linked immunosorbent assay (ELISA) assays

We used commercially available solid-phase ELISA methods for TSP-1, endostatin, and Ang-2 according to assay protocol developed by the manufacturer (R&D Systems, Minneapolis, MN). The plates were analyzed using the microplate reader Victor 2 Multilabel Counter (Wallac, Turku, Finland) at wavelength 450 nm. Concentrations were reported as ng/mL or pg/mL, respectively.

### 2.6 Statistical analysis

Continuous variables were tested for normal distribution using the Kolmogorov-Smirnov test; and the continuous data with normal distribution were expressed as mean ± standard deviation, while other data were given as median. The categorical variables were defined as percentage. Student *t* test or Mann–Whitney *U* test was used for the univariate analysis of the continuous variables and the χ^2^ test for the categorical variables.^[10]^ Mean values were compared by analysis of variance (ANOVA) among different groups. Pairwise comparison of continuous variables after ANOVA test was done using post hoc least significant difference test. Logistic regression with Enter method was used for multivariate analysis of independent variables. In this model, coronary collateral development (poor/good) was the dependent variable, while variables such as endostatin, TSP-1, male gender, history of hypertension/diabetes/hyperlipidemia, prior smoking, previous MI, and LVEF were the covariates. Besides endostatin and TSP-1, the variables chosen were based on potential covariates of coronary collateral formation reported previously^[12]^ and those used in published research work.^[10]^ The multiple linear regression was used to evaluate the relationship between endostatin and different factors in a generalized linear model. The following variables as determined by previous studies^[13,14]^ were included in this analysis: male gender, age, history of hypertension/diabetes/hyperlipidemia, prior smoking, previous MI, collateral grade, LVEF, HbA1c, and eGFR. All tests of significance were 2-tailed. A *P* value < 0.05 was considered as statistically significant. All statistical analyses were performed using the statistical package SPSS for Windows (Version 15.0, SPSS, Chicago, IL).

## 3 Results

### 3.1 Patient characteristics

We enrolled 149 patients in this study, and their clinical characteristics are shown in Table 1. One hundred and ten patients had at least 1 CTO lesion confirmed by CAG, of whom 31 were divided into poor collateral group, while 79 into good collateral group. Compared with control group, poor collateral CTO group included patients with elevated NT-proBNP (326.7 vs 70.7 pg/mL, *P* = 0.001) and worse LVEF (54.9 ± 13.8% vs 64.1 ± 9.3%, *P* = 0.001), while good collateral CTO group included more male patients (84.8% vs 64.1%, *P* = 0.011). However, there is no gender difference between good and poor collateral group. Compared with poor collateral CTO group, good collateral CTO group had less NT-proBNP (183.0 vs 326.7 pg/mL, *P* = 0.049), better LVEF (60.7 ± 9.9% vs 54.9 ± 13.8%, *P* = 0.013), more RCA CTO lesions (51.9% vs 16.1%, *P* = 0.001) and less left circumflex artery (LCX) CTO lesions (24.1% vs 45.2%, *P* = 0.03).

**Table 1:**
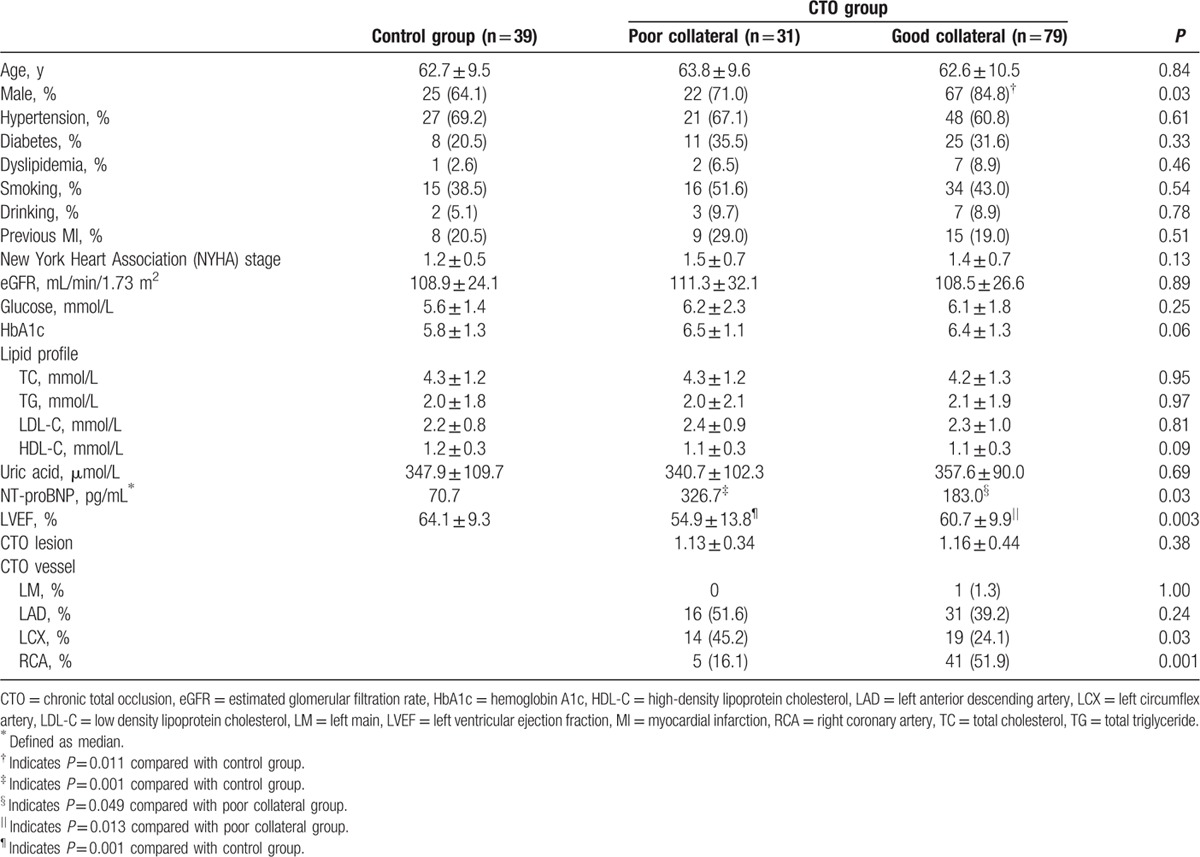
Baseline characteristics of the study population.

### 3.2 Serum TSP-1, endostatin, and Ang-2 level

Serum TSP-1 level was lower in CTO with poor collateral group (290.7 ± 157.8 vs 821.4 ± 638.3 ng/mL, *P* = 0.01) and good collateral group (419.3 ± 374.6 vs 821.4 ± 638.3 ng/mL, *P* = 0.015) compared with control group. However, no difference in TSP-1 level was detected between poor and good collateral group. The serum endostatin level was significantly higher in poor collateral CTO group compared with control group and good collateral CTO group, respectively (96.2 ± 30.4 vs 77.8 ± 16.5 ng/mL, *P* = 0.007; 96.2 ± 30.4 vs 81.2 ± 30.4 ng/mL, *P* = 0.018). The serum Ang-2 level did not show a significant difference among 3 groups (Table 2).

**Table 2:**
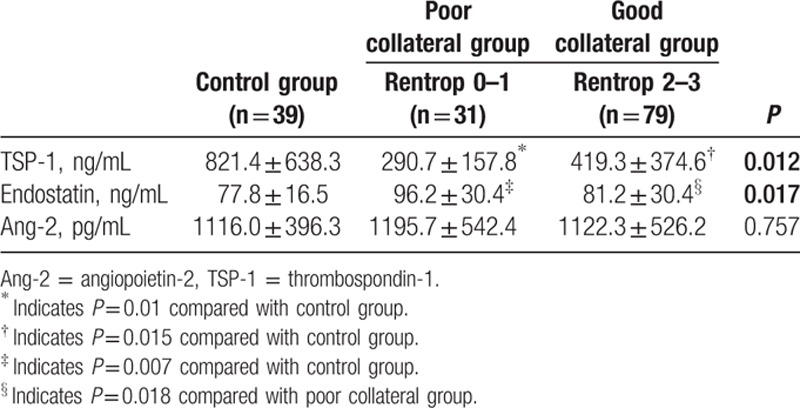
TSP-1, endostatin and Ang-2 levels among the groups.

### 3.3 Determinants of good collaterals

To determine whether specific angiostatic factors and clinical characteristics are predictive of good collateralization, we performed a multivariate analysis by using logistic regression and found that only decreased endostatin (*P* = 0.027) and male gender (*P* = 0.048) were independently related to good coronary collateral development (Table 3).

**Table 3:**
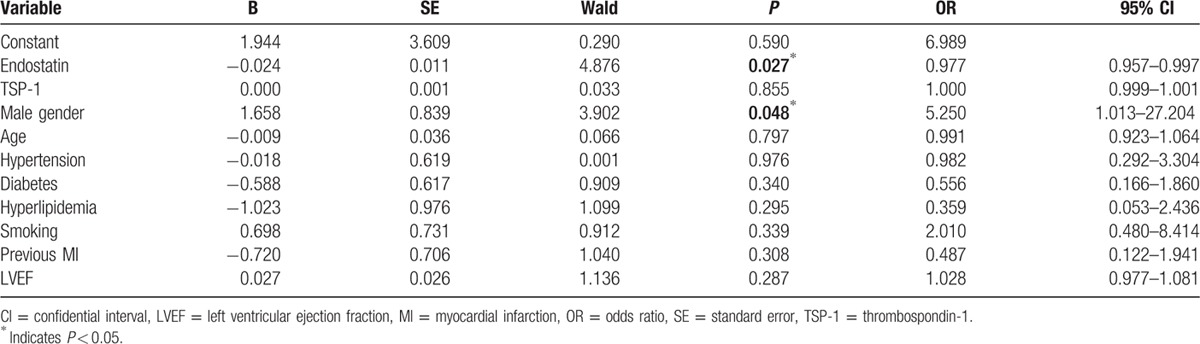
Associations between different factors and good coronary collateral development by using the logistic regression.

### 3.4 Association of endostatin and different factors

In the multiple linear regression model, history of diabetes, collateral grade, and eGFR were correlated to serum endostatin levels (Table 4).

**Table 4:**
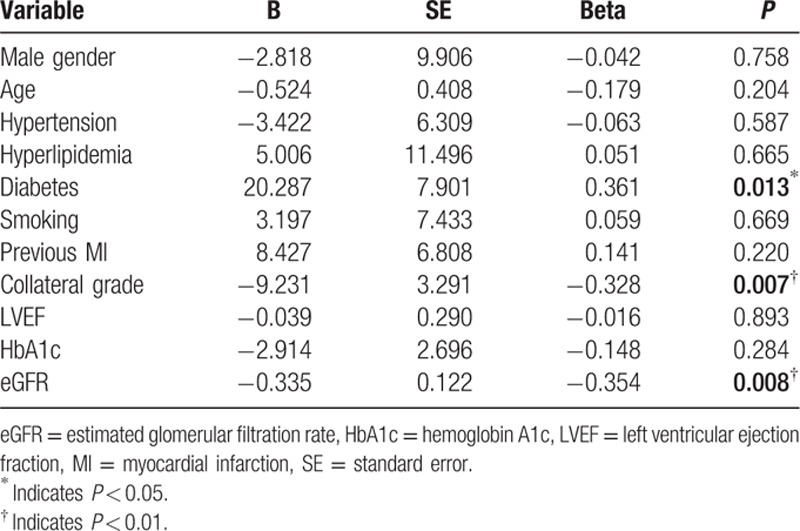
Associations between different factors and endostatin by using the multiple linear regression.

## 4 Discussion

To our knowledge, the present study interrogated whether coronary collateral development has any association with serum angiostatic cytokines-TSP-1, endostatin, or angiogenic cytokine-Ang-2 in patients with CTO and identified for the first time that decreased venous endostatin was an independent predictor of good coronary collateral development. Furthermore, the serum endostatin level is associated with history of diabetes, collateral grade, and eGFR.

In the study, we found that good collateral flow is more frequent in those with RCA occlusion compared with those with left coronary artery occlusion. This has been identified by other researchers not only under the condition of acute^[15]^ but also chronic vessel occlusion.^[16]^ Stadius ML speculated that low resistance in the RCA bed may be the reason for this. The left coronary arteries serve only left ventricular myocardium, a relatively high-resistance bed, while the RCA, on the other hand, serves both left and right ventricular myocardium and the latter is a relatively low-resistance bed. This may well explain why collateral circulation is more common with RCA than with left anterior descending artery or LCX occlusion.^[17]^

The degree of coronary collateral development varies among patients with ischemic cardiac disease. The factors affecting functional coronary collateral formation have not been described very well.^[18]^ Investigation of key cytokines and mechanisms affecting collateral development is crucial to develop new strategies to enhance collateral blood supply in patients who are not suitable for mechanical revascularization. Cytokines related to human coronary collateral formation identified by previous clinical studies included angiogenic factors such as VEGF, erythropoietin,^[10]^ HGF,^[19]^ basic fibroblast growth factor,^[20]^ monocyte chemotactic protein-1,^[21]^ macrophage inhibitory cytokine-1,^[22]^ and angiostatic factors such as angiostatin,^[23]^ TSP-1,^[11]^ and endostatin.^[24]^ However, stimulation of collateral vessel development by angiogenic growth factor (VEGF and FGF) therapy generated disappointing efficacy outcomes in late-stage clinical trials.^[25]^ The exact reasons for this have not been determined yet. Perhaps a switch of focus from angiogenic to angiostatic factors will help to get better mechanistic understanding of adaptive vascular responses in the heart, which may favor therapeutic angiogenesis in the future. Besides, exploration for new angiogenic cytokines which play dominant role in stimulating collateral formation may also be a priority.

It has been confirmed that antiangiogenic cytokines such as angiostatin, TSP-1, and endostatin played a part in human coronary collateral formation. Matsunaga et al^[23]^ reported a negative relationship between serum angiostatin and collateral grade. Recently, a study from our group demonstrated a paradoxical higher level of TSP-1 in patients with good collaterals compared with poor collaterals, which implied a self-adjustment mechanism in patients with poor collaterals to favor collateral formation.^[11]^ In the present study, the same trend was observed among 3 groups; however, no statistical significance of serum TSP-1 level was detected between good and poor collaterals group perhaps due to a different group method. Besides, logistic regression analysis did not identify TSP-1's relationship with collateral grade. It suggested that TSP-1 may not be a dominant factor in coronary collateral formation and is less qualified as a marker for collateral formation.

Endostatin, the 20-kDa cleavage product of collagen XVIII, has been shown to be a potent inhibitor of angiogenesis by inhibiting endothelial cell migration, proliferation, and inducing endothelial cell apoptosis.^[26]^ Endostatin is elevated in patients with diabetes^[13]^ and chronic kidney disease,^[14]^ which is consistent with our data that endostatin is positively related to diabetes, while negatively related to eGFR. Previous study demonstrated that endostatin level in coronary sinus^[27]^ and human heart tissue^[13]^ was elevated in patients with CAD. Besides, increased production of endostatin within the coronary circulation as shown by elevated coronary sinus-left ventricle (CS-LV) endostatin gradient has been confirmed in patients with severe CAD compared with moderate coronary stenosis.^[27]^ Other studies which focused on the relationship between endostatin and coronary collaterals identified that pericardial fluid endostatin level was significantly higher in patients with no collaterals compared with Rentrop grade 3 coronary collaterals.^[24]^ There is a tendency of more endostatin production within the coronary circulation as shown by CS-LV gradient in patients with poorly developed collaterals than in those with well developed collaterals.^[27]^ However, statistical significance was not reached in this study perhaps because of small sample size. To our knowledge, our study was the first to investigate venous serum endostatin level in patients with CTO with a large sample size. We demonstrated a relationship between endostatin and coronary collateral grade and also identified decreased serum endostatin as an independent predictor of good coronary collateral development in patients with CTO. As a result, we hypothesized that repression of angiostatic cytokine, such as endostatin, may to some extent induce angiogenesis in patients with ischemic heart disease. Perhaps regulation of angiostatic factors will be a new approach in stimulation of angiogenesis in the future.

To explore new angiogenic target, we choose to study the relationship between serum Ang-2 and collateral grade. Ang-2 modulates endothelial cell biology, destabilizes blood vessels, and promotes VEGF-induced neovascularization to facilitate angiogenesis. It acts differently from Ang-1 which is characterized by vessel stabilization.^[28]^ In adult humans, Ang-2 is expressed only at sites of vascular remodeling, so circulating levels of Ang-2 may acutely reflect the vascular regeneration and repair.^[29]^ However, in our study, no relationship between venous serum Ang-2 level and collateral grade was detected. Similar results were reported by Mitsuma et al in a quite smaller group of patients. No difference of Ang-2 in coronary circulation as shown by CS-LV gradient was detected between patients with good and poor collaterals. However, this study identified Tie-2, the receptor of Ang-1 and Ang-2, as a crucial factor in the development or maintenance of coronary collateral vessels in severe CAD as more production of Tie-2 within the coronary circulation was related to well developed coronary collateral.^[30]^ As a result, further study on the Ang/Tie-2 system is necessary for exploration of new therapeutic target for coronary angiogenesis.

### 4.1 Limitation

First, several growth factors and cytokines are produced locally in ischemic cardiac tissue and they are lower in the systemic circulation because of dilution upon washout.^[31]^ In this study, we used venous samples which may not necessarily demonstrate local cardiac concentration. Second, the association observed between endostatin and collateral formation cannot be categorized as causative. Future studies are needed to investigate if the inhibition of endostatin may improve collateral formation and cardiac function.

## 5 Conclusion

Lower serum endostatin level was related to good coronary collateral development in patients with CTO. Circulatory endostatin may be a useful biomarker for coronary collateral development and potential target for therapeutic angiogenesis in patients with CTO. Further study with inhibition of endostatin in ischemic myocardium is necessary to prove the role and mechanism it played in myocardial angiogenesis.
